# Determinants of gender disparities in psychological distress in the South African population aged 15 years and older: Findings from the 2017 National HIV prevalence, incidence, behaviour, and communication survey

**DOI:** 10.1371/journal.pmen.0000220

**Published:** 2025-05-27

**Authors:** Nompumelelo P. Zungu, Musawenkosi Mabaso, Tawanda Makusha, Lehlogonolo Makola, Ronel Sewpaul, Olive Shisana

**Affiliations:** 1 Public Health, Societies and Belonging Division, Human Sciences Research Council, Pretoria, South Africa; 2 School of Nursing and Public Health, University of KwaZulu-Natal, Durban, South Africa; 3 Clinical Research Department, Africa Health Research Institute, Durban, South Africa; 4 Population and Development Studies, School of Built Environment and Development Studies, University of KwaZulu-Natal,; 5 Department of Psychiatry and Mental Health, University of Cape Town, Cape Town, South Africa; UCL: University College London, UNITED KINGDOM OF GREAT BRITAIN AND NORTHERN IRELAND

## Abstract

Psychological distress, characterized by symptoms such as anxiety, depression, and emotional suffering, is a major public health issue with well-documented gender disparities. This study examined the determinants of gender differences in psychological distress among South Africans aged 15 years and older using data from the 2017 South African National HIV Prevalence, Incidence, Behaviour, and Communication Survey. The cross-sectional survey employed a multi-stage stratified random cluster sampling design. Psychological distress was measured using the Kessler 10-item Psychological Distress Scale (K10), where a score of ≥20 indicated some level of distress. Of the 8,148 participants, the weighted prevalence of psychological distress was 19.3% (95% CI: 17.8–20.9), with a significantly higher prevalence among females (22.2%, 95% CI: 20.2–24.4) than males (16.4%, 95% CI: 14.4–18.6). Multivariable backward stepwise logistic regression models were used to identify factors associated with psychological distress for each gender, and adjusted odds ratios (AORs) with 95% confidence intervals (CIs) were reported. Among males, higher odds of distress were associated with fair/poor self-rated health (AOR = 1.7, 95% CI: 1.2–2.4) and excessive alcohol use (AOR = 1.6, 95% CI: 1.1–2.3). Protective factors included tertiary education (AOR = 0.5, 95% CI: 0.3–0.9), residence in rural formal/farm areas (AOR = 0.6, 95% CI: 0.4–1.0), and being HIV negative (AOR = 0.7, 95% CI: 0.4–1.0). For females, distress was significantly associated with fair/poor self-rated health (AOR = 2.6, 95% CI: 2.0–3.4) and excessive alcohol use (AOR = 2.0, 95% CI: 1.3–3.1). Lower odds were found among the employed (AOR = 0.7, 95% CI: 0.5–0.9), residents of rural informal/tribal (AOR = 0.6, 95% CI: 0.5–0.8) and rural formal/farm areas (AOR = 0.6, 95% CI: 0.4–0.9), and those with accurate HIV knowledge and myth rejection (AOR = 0.6, 95% CI: 0.4–0.7). The findings emphasize the need for gender-specific mental health interventions targeting modifiable risk factors to reduce psychological distress in South Africa.

## Introduction

Psychological distress broadly defined as a state of emotional suffering characterized by symptoms of depression (e.g., loss of interest; unhappiness; desperateness) and anxiety (e.g., restlessness; feeling tense) is a growing public health concern affecting the quality of life, work productivity, and life expectancy in a given population [1,2]. It is also characterized by sleep disturbance, fluctuating eating patterns, headaches, excessive tiredness, forgetfulness, memory, and behavioral problems [1,2]. Anxiety and depression are a major contributor to the burden of disease in all regions of the world [3,4]. However, in sub-Saharan Africa data on the prevalence of psychological distress is limited [5]. In South Africa, small-scale studies have found a high prevalence rate of psychological distress in the general population [6–8]. Several factors are known to influence the development of anxiety and depression, and these include a combination of demographic characteristics, biological factors, and socio-economic status [3,9,10].

Population surveys and epidemiological studies in several countries indicate that women report higher levels of psychological distress than men [11–15]. Differences in psychological distress between women and men impact health status and the overall disease burden and contribute to disparities in communicable diseases such as tuberculosis (TB) and HIV [16–19] and non-communicable diseases such as cardiovascular disease, diabetes, and hypertension [20–22]. Disparities in psychological distress between women and men were also observed during the outbreak of the COVID-19 pandemic globally [23–25].

Evidence shows that biological factors (sex hormones and differential hormonal stress response) interact with psychosocial factors to produce differences in psychological distress between women and men [20]. Disparities in psychological distress between men and women vary according to context or socio-cultural setting [1–3,6,9]. The observed differences are, to a large extent, influenced by socially defined roles and behavioral norms attributed to women and men and unequal interpersonal relationships justified by social norms linked to socio-demographic characteristics such as age, population group, educational attainment, and type of settlement/residential area [12,13].

Other studies have found that social inequalities partly explained differences in psychological distress between women and men, with higher levels found in those from the lowest social class compared to the highest class [10]. This was also evidenced during the outbreak of the COVID-19 pandemic, where psychological distress was also differentially associated with social class differences between men and women [23–25]. Others found that gender differences in psychological distress decrease or eventually disappear when women and men have similar socioeconomic conditions [11]. Therefore, biological, social, demographic, and structural factors interact to determine differences in psychological distress between women and men.

Consequently, women and men may have different treatment needs, ranging from medication doses and types to behavioral and psychosocial treatments [8]. Therefore, intervention strategies could benefit from an improved understanding of the critical determinants that lead to psychological distress and how these may differ between women and men. However, the prevalence of psychological distress and associated factors vary across different population groups depending on the setting and context [14–18]. As a result of the differences in determinants of psychological distress by gender in other populations that have been identified in the literature [14–18] the current study investigates the determinants of psychological distress in adolescent and adult women and men, in the South Africa context using household-based population survey data.

This study is guided by the social determinants of health framework, which is a multi-level analytical approach used to understand multiple factors contributing to psychological distress [14,19–22]. The framework is responsible for explaining health inequalities and disparities across population groups based on the theory that various social and economic factors such as gender, age, education, marital status, and employment can influence an individual’s exposure to stressors. The analysis examines the association of psychological stress with demographic, socio-behavioral, and health-related factors including HIV-related variables. We hypothesized that the factors associated with psychological distress differed between women and men.

## Methodology

### Ethical approval

The survey protocol was approved by the HSRC’s Research Ethics Committee (REC: 4/18/11/15) and the Associate Director for Science, Center for Global Health, Centers for Disease Control and Prevention (CDC), GA, USA. Informed consent was sought from all participants aged 18 years and older while assent was obtained from the youth aged 15–17 years after seeking consent from their parents/guardians.

### Study design and population

This paper used cross-sectional data obtained from the 2017 National HIV Prevalence, Incidence, Behaviour, and Communication Survey, conducted using a multi-stage stratified random cluster sampling design, described in detail elsewhere [26]. The survey used a systematic probability sample of 15 households drawn randomly from 1000 small area layers (SALs) selected from 84 907 SALs released by Statistics South Africa in 2015 [27]. The sampling of SALs was stratified by province, locality type, and race group. This was a population-based household survey where all members of selected households were invited to participate in the survey. Consequently, the survey population excluded people living in institutions such as old-age homes, orphanage homes, homeless people who live on the streets or in shelters, student residences, and uniformed-service barracks. In addition, the exclusion criteria were also based on whether people were able to provide clear consent according to their understanding and comprehension of the consent process and the information provided. The sampling frame is similar to that implemented in the previous four surveys.

The survey targeted 15,000 visiting points (VP) but only 12,435 of these (82.9%) were approached. Among the 12,435 VPs, only 11,776 (94.7%) were valid households. Of the 11,776 valid VPs, 9,656 VPs agreed to participate in the survey at the household level. This translates to a household response rate of 82%. All consenting members of the selected households formed the ultimate sampling unit. This included household members of all ages who resided in selected households.

One household questionnaire, and questionnaires for parents/guardians of children aged 0–11 years, children aged 12–14 years, and persons aged 15 years and older were administered. The survey solicited information on socio-demographic characteristics, HIV-related knowledge, attitudes, practices, sexual history, and risky sexual behaviors as well as health-related information including mental health. The questionnaires were fieldworker administered and electronically captured using CSPro software on Mercer tablets. In addition, dried blood spots (DBS) specimens were also collected from participants who consented to laboratory testing for HIV, viral load, exposure to ARVs, HIV drug resistance, and the limiting antigen (LAg) avidity assayed in incidence estimation [26].

Within the 9,656 VPs that agreed to participate in the survey at the household level, 8,184 individuals aged 15 years and older responded to questions on experiences of anxiety and depressive disorders, which were used to measure psychological distress.

## Measures

### Dependent variable

The primary outcome variable, psychological distress, was derived from respondents’ experiences of anxiety and depressive disorders measured using the 10-item Kessler Psychological Distress Scale (K10) [28]. The ten-item questionnaire assesses symptoms commonly associated with depression and anxiety by asking: *‘In the past 30 days how often did you feel: Tired out for no good reason? So nervous that nothing could calm you down? Hopeless; Restless or fidgety: So restless that you could not sit still; Depressed? That everything was an effort? So sad that nothing could cheer you up? Worthless?’.* Each response is assessed on a 5-point Likert scale from 0 to 4, with increasing values corresponding to higher levels of distress. A total score is derived by summing all items, ranging from 0–40. The scores from these responses were then summed to calculate a total score indicating whether the respondents were likely to experience psychological distress, that is, likely to be well (score below 20), experiencing mild (score 20–24), moderate (score 25–29) or severe (score 30 and above) psychological distress. The scores were then dichotomized into a binary outcome; those who scored <19 absence of psychological distress = 0) and those who scored ≥20 (presence of mild, moderate, or severe psychological distress = 1).

### Independent variables

Explanatory variables included socio-demographic variables such as age categories in years (15–24, 25–49, 50 years and older), self-reported gender (Male and Female), race (Black African and Other, including Coloured, Indian and White), marital status (Married, Single or Never Married, which exclude divorced/separated and widower/widow), educational level (Primary/no education, Secondary and Tertiary), employment status (Unemployed and Employed), and locality type (Urban, Rural informal/tribal areas, Rural formal/farm areas). Socio-behavioral variables included having experienced physical intimate partner violence (No and Yes), excessive alcohol use (measured using the Alcohol Use Disorder Identification Test (AUDIT) [29,30], dichotomized into No (0 = Abstainers/non-hazardous alcohol use; and 1 = Excessive alcohol use which includes high-risk drinkers and hazardous drinkers), a health-related variable, self-rated health (Fair/poor and Good/excellent) and HIV-related variables such as the perceived risk of HIV infection (Low and High), HIV serostatus (Positive and Negative), and correct HIV knowledge and myth rejection about HIV transmission and prevention (No and Yes) from UNAIDS [31] based on responses to the questions of whether *“AIDS can be cured; a person reduces the risk of HIV by having fewer sexual partners; a healthy-looking person have HIV; a person gets HIV by sharing food with someone who is infected; and a person reduces the risk of getting HIV by using a condom every time he/she has sex”*.

### Statistical analysis

The data was weighted to account for the complex multilevel unequal sampling probabilities in the survey design. Descriptive statistics (frequencies and percentages) were used to summarize characteristics of the study population and psychological distress by gender. Differences between categorical variables were assessed using Pearson’s chi-squared test. A multivariable backward stepwise logistic regression selection model was fitted to determine the factors associated with psychological distress among males and females aged 15 years and older. The “svy” command was used to take into account the complex survey design in the analysis. The study presents weighted and therefore population representative prevalence estimates of psychological distress. Adjusted Odds Ratios (AOR) with 95% Confidence Intervals (CI) and p-value less than 0.05 was used to determine the magnitude and direction of the relationship and the level of statistical significance. All analyses were conducted using STATA Statistical Software Release 15.0 [32].

## Results

### Characteristics of the study population

The study consisted of 8,184 participants aged 15 years and older who responded to questions on experiences of anxiety and depressive disorders and submitted a blood sample for biomarker testing (Table 1). The study population consisted of 49.8% males (n = 3,337) and 50.2% females (n = 4,847). Most study participants were aged 25–49 years, were Black African, were never married, had secondary education, were unemployed, and resided in urban areas. Overall, just above 13.5% of study participants used alcohol excessively. About a fifth rated their health as fair/poor and perceived themselves as at high risk of HIV infection. Just over a fifth of study participants were HIV positive, and over a third had correct knowledge and myth rejection of HIV transmission and prevention.

**Table 1:** Socio-demographic characteristics of the study population (n = 8 184).

Variables	Study population		Males		Females	
**Age groups (years)**	**Total**	**%**	**n**	**%**	**n**	**%**
15–24	1 371	15.3	602	15.5	769	15.0
25–49	4 460	61.6	1 807	63.2	2 653	59.9
50 and older	2 353	23.2	928	21.2	1 425	25.1
**Race groups**						
African	6 082	79.0	2 463	79.1	3 619	79.0
Other	2 102	21.0	874	20.9	1 228	21.0
**Marital status**						
Married	2 712	37.1	1 212	36.8	1 500	37.4
Never married	4 480	62.9	1 880	63.2	2 600	62.6
**Education level**						
No education/Primary*	1 302	15.8	502	15.2	800	16.3
Secondary	4 401	66.4	1 783	66.5	2 618	66.3
Tertiary	1 030	17.8	458	18.3	572	17.4
**Employment status**						
Unemployed	4 923	56.3	1 629	46.4	3 294	66.0
Employed	3 140	43.7	1 656	53.6	1 484	34.0
**Locality type**						
Urban areas	5 121	71.9	2 150	74.3	2 971	69.5
Rural informal/tribal areas	2 278	23.0	769	19.5	1 509	26.4
Rural/farm areas	785	5.2	418	6.2	367	4.2
**Physical IPV**						
No	6 960	89.5	2 968	92.7	3 992	86.4
Yes	774	10.5	199	7.3	575	13.6
**Excessive alcohol use**						
No	6 675	86.5	2 476	78.5	4 199	94.6
Yes	785	13.5	550	21.5	235	5.4
**Self-rated health**						
Excellent/good	6 587	81.5	2750	82.4	3837	80.6
Fair/poor	1 594	18.5	587	17.6	1007	19.4
**Self-perceived risk of HIV**						
Low	6 043	80.8	2572	81.6	3471	79.8
High	1 282	19.2	527	18.4	755	20.2
**HIV serostatus**						
Positive	1 262	21.5	329	16.4	933	26.2
Negative	4 448	78.5	1 866	83.6	2 582	73.8
**Correct HIV knowledge and myth** **Rejection**						
No	5 076	62.9	2 062	63.3	3 014	62.6
Yes	3 099	37.1	1270	36.7	1 829	37.4

The table presents weighted proportions. No education/Primary combined due to small counts for no education variable. Subtotals do not add up to the overall total due to non-response and/or missing data. IPV– Intimate Partner Violence.

### Prevalence of psychological distress

Of 8,148 participants, the weighted prevalence of psychological distress was 19.3% [95% CI: 17.8-20.9, n = 1 532]. The prevalence of psychological distress was significantly higher (p < 0.001) among females (22.2%, 95% CI: 20.2-24.4) compared to males (16.4%, 95% CI: 14.4-18.6). Table 2 shows the prevalence of psychological distress by socio-demographic characteristics and gender. Among males, the prevalence of psychological distress was significantly higher among Black Africans, those who never married, those with no education/primary education level, and the unemployed. There were no differences in psychological distress by age among females, yet there were differences by age among males. Among females, the prevalence of psychological distress was significantly higher among Black Africans, those who were never married, those with no education/primary education level, the unemployed, and those from urban areas.

**Table 2:** Prevalence of psychological distress by socio-demographic characteristics and gender.

Variables	Study population	Males			Females		
	Total	%	95% CI	p-value	%	95% CI	p-value
**Age (years)**				0.582			0.746
15–24	1 371	14.4	10.8-18.9		23.6	18.2-29.9	
25–49	4 460	16.9	14.3-19.9		21.7	19.4-24.2	
50+	2 353	16.2	13.0-19.9		22.7	19.3-26.4	
**Race groups**				<0.001			<0.001
African	6 082	18.3	15.9-20.9		24.1	21.7-26.7	
Other	2 102	9.1	6.6-12.2		15.1	12.0-18.8	
**Marital status**				0.018			0.003
Married	2 712	13.4	10.7-16.6		17.6	14.8-20.8	
Never married	4 480	18.1	15.5-21.0		24.0	21.1-27.1	
**Education level**				0.003			0.047
No education/Primary	1 302	18.9	13.9-25.1		24.3	20.1-29.2	
Secondary	4 401	18.1	15.4-21.1		23.4	20.7-26.4	
Tertiary	1 030	9.1	6.0-13.6		16.7	12.3-22.2	
**Employment status**				0.008			<0.001
Unemployed	4 923	19.1	16.4-22.1		24.8	22.3-27.6	
Employed	3 140	14.2	11.7-17.1		17.2	14.3-20.5	
**Locality type**				0.183			0.122
Urban areas	5 121	16.6	14.2-19.4		23.2	20.5-26.1	
Rural informal/tribal areas	2 278	17.0	13.5-21.3		20.6	17.5-24.2	
Rural/farm areas	785	10.7	7.5-15.1		15.7	10.7-22.4	
**IPV Physical**				0.086			0.027
No	6 960	15.7	13.7-17.9		21.2	19.0-23.5	
Yes	774	24.0	14.8-36.6		27.7	22.1-34.1	
**Excessive alcohol use**				0.002			0.007
No	6 675	14.3	12.2-16.6		21.5	19.4-23.8	
Yes	785	22.0	17.5-27.3		32.7	24.4-42.3	
**Self-rated health**				<0.001			<0.001
Excellent/good	6 587	13.7	11.7-16.0		17.7	15.8-19.9	
Fair/poor	1594	28.6	23.1-34.9		40.8	36.1-45.8	
**Self-perceived risk of HIV**				0.034			0.012
Low	6 043	14.3	12.2-16.7		19.9	17.6-22.3	
High	1282	19.6	15.0-25.1		26.1	21.7-31.1	
**HIV serostatus**				0.001			0.150
Positive	1 262	27.0	20.3-34.9		26.7	22.4-31.6	
Negative	4 448	15.6	13.3-18.2		23.2	20.6-26.1	
**Correct HIV knowledge and myth rejection**				<0.001			<0.001
No	5 076	19.1	16.4-22.0		25.8	23.3-28.5	
Yes	3 099	11.7	9.3-14.8		16.2	13.4-19.3	

Among males, the prevalence of psychological distress was significantly higher among those who reported excessive alcohol use, those who reported fair/poor self-rated health, those who perceived themselves as being at high risk of HIV infection, those who were HIV positive, and those with incorrect HIV knowledge and myth rejection. Among females, the prevalence of psychological distress was significantly higher among those who reported IPV, excessive alcohol use, those who reported fair/poor self-rated health, those who perceived themselves as at high risk of HIV infection, and those with no correct HIV knowledge and myth rejection.

The table presents weighted row proportions of the presence relative to the absence of psychological distress. No education/Primary combined due to small counts for no education variable. Subtotals do not add up to the overall total due to non-response and/or missing data. IPV– Intimate Partner Violence. CI – confidence intervals. Peason’s Chi-square test was used to compare differences between categorical variables.

### Determinants of psychological distress

**Fig 1:**
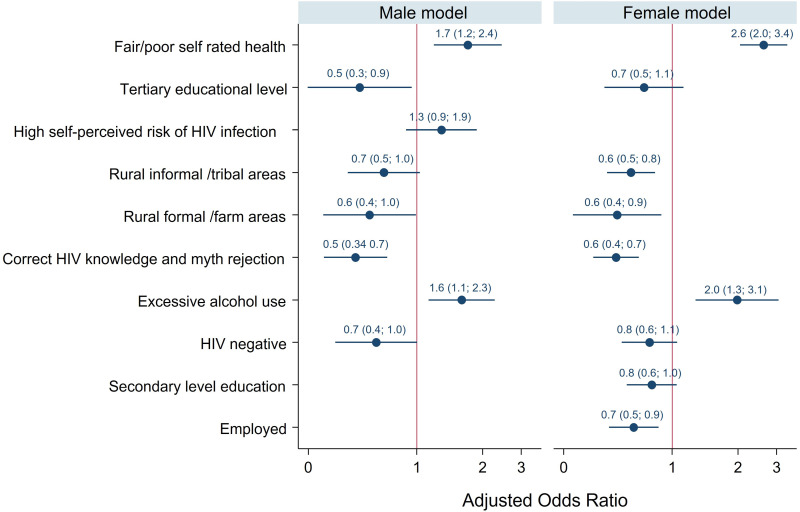
Coefficient plot of the multivariable logistic regression models for the determinants of psychological distress among South Africans aged 15 years and older by gender, showing adjusted odds ratios for all variables selected in the final models, confidence intervals, and a line of no effect.

Fig 1 shows adjusted odds ratios of the relationship between psychological distress and all covariates selected in the final male and female multivariable logistic regression models. In the male model, the odds of psychological distress were significantly higher among those who reported fair/poor self-rated health than excellent/good self-rated health and those who reported excessive alcohol use than those with low-risk alcohol use or abstainers.

The odds of psychological distress were significantly lower among those with tertiary education than those with no education/primary education level, those from rural formal/farm areas than those from urban areas, those with correct HIV knowledge and myth rejection, and those who were HIV negative than HIV positive individuals.

In the female model, the odds of psychological distress were significantly higher among those who reported fair/poor self-rated health than excellent/good self-rated health and those with excessive alcohol use than those with low-risk alcohol use or who were abstainers. The odds of reporting psychological distress were significantly lower among the employed than unemployed, those who reside in rural informal/tribal areas than in urban areas, those from rural formal/farm areas than urban areas, and those with correct HIV knowledge and myth rejection.

## Discussion

This nationally representative survey revealed that 19.3% of the study population reported psychological distress. The estimated prevalence of psychological distress was lower than what has been reported in other large population-based studies in South Africa. Their estimates ranged between 22% and 35% [33,34]. The differences observed may be due to the sub-populations used to assess psychological distress. Similar to current findings, the results of other studies indicate that females had a higher prevalence of psychological distress than males [35–39]. Furthermore, in line with other studies, the prevalence of psychological distress varied by socio-demographic factors such as race/ethnicity, level of education, marital status, socioeconomic status, occupation, substance use, and geographic location [40–43] as well as by health and HIV-related factors [44–48].

This study identified social determinants of mental health that were common for both males and females. Psychological distress was associated with fair/poor self-rated health, urban residence, and hazardous alcohol use among both males and females; findings which have been confirmed in both genders in other studies. Furthermore, correct knowledge and rejection of myths relating to HIV transmission and prevention were protective of psychological distress in both males and females in this study. Gender disparities in the social determinants of health were also found, where being HIV-negative and having tertiary education was protective of psychological distress in males but not females, and where employment was protective of psychological distress in females and not males.

The finding that psychological distress was associated with fair/poor self-rated health for both males and females is consistent with other studies [44,49,50]. Studies show that people experiencing psychological distress are likely to suffer from poor general physical health due to negative mental health attributes related to, among others, restlessness or sleeplessness and worrying or feeling unhappy [51,52]. Poor health can also negatively impact psychological well-being, and the association can be bidirectional [44,47,53]. A better understanding of the link between self-reported health status and drivers of psychological distress may be crucial to averting additional complexities associated with both conditions [51].

As previously observed in other studies, the models showed that psychological distress was associated with excessive alcohol use [40–42]. Literature shows that the two are connected as alcohol use disorder is in itself a mental disorder [54–56]. Conversely, excessive alcohol use is often triggered by psychological distress as a coping mechanism [54–56]. However, the effect of modification of alcohol use on psychological distress is not known.

The finding that living in urban areas was associated with an increased likelihood of psychological distress has also been observed in other studies [43,44]. This has been attributed to social distress linked to living in an urban environment, such as violence and crime, including social disparities and stressors such as low levels of education, unemployment, and low income [43,44]. However, there have been few studies conducted on the relationship between locality type/place of residence and psychological distress, with conflicting evidence [44]. Some studies found no such evidence, while others showed that urban areas were more prone to higher levels of distress than rural areas and vice versa [43,44]. Therefore, more studies are needed specifically dealing with the relationship between locality type/place of residence and psychological distress. The finding that psychological distress was lower in males and females who had correct knowledge and rejection of myths relating to HIV transmission and prevention has not been confirmed previously and may warrant further investigation. However, correct knowledge and myth rejection may reflect higher health literacy and education levels, which were linked to lower psychological distress.

In congruence with the current findings, evidence shows that, in sub-Saharan Africa, HIV-positive individuals have higher psychological distress compared to the general population [46]. Studies show that most HIV-positive individuals who experience high distress were newly diagnosed individuals who were exposed to multiple stressors, which include concerns of confidentiality, discrimination/stigmatization, disclosure, and fear of infecting others [19,57]. Furthermore, literature shows that the level of psychological distress decreases over time due to the development of positive thinking and other coping mechanisms [45,58]. In the current study, the period of HIV diagnosis and levels of psychological distress were not investigated and could be focused on in future research.

The link between unemployment and psychological distress, especially among females, is well-documented in the literature [59–61]. Generally, being unemployed increases the probability of experiencing stress-inducing factors such as socioeconomic deprivation, lack of resources, limited opportunities, and low self-regard [59,60]. Gender differences and the effect of unemployment on mental health are related to the different social positions and roles associated with psychosocial and economic needs depending on the context, such that unemployed women have worse mental health and lower life satisfaction than unemployed men in some settings [61–63].

### Study limitations

The data used in the analysis are based on a self-report questionnaire and may be affected by nonresponse bias, recall bias, and social desirability bias. This study includes complete case analysis and although it yields unbiased estimates when data are missing completely at random, such analyses can be biased if data are missing by certain mechanisms that depend on observed or unobserved variables. Missing data on covariates is often imputed using multiple imputations with chained equations to resolve bias associated with deleting cases with missing data. The cross-sectional design is limited to assessing the association between psychological distress and potential covariates and cannot infer causality. Though costly to set up and maintain for a long period, South Africa should consider longitudinal studies because they allow researchers to understand disease progression, track the effects of interventions over time, and identify risk factors, ultimately informing better prevention and care strategies. Control for confounding was limited to the questions asked by the survey, and there may be other covariates related to gender differences in psychological distress that were not examined in the study. The complex interplay between psychological distress and covariates was not explored. Future analysis should consider mediator and moderator analyses to explore pathways to experiencing psychological distress. Despite these limitations, the strength of the study is that it is nationally representative, and therefore generalizable to South Africans aged 15 years and older. In addition, this study contributes to the literature toward a better understanding of factors associated with psychological distress.

## Conclusion

The findings of this study showed that socio-demographic, socio-behavioral, and health-related factors were independently associated with the presence of psychological distress in the study population. The study showed that an increased likelihood of psychological distress was associated with fair/poor self-rated health, alcohol misuse, and residing in urban areas in both males and females and reporting no correct HIV knowledge and myth rejection. Among males, lack of education/low educational attainment and HIV-positive serostatus were associated with increased psychological distress. Among females, unemployment was associated with increased psychological distress. Understanding these determinants is vital to crafting targeted interventions and fostering an environment that promotes psychological well-being for all individuals. Gender-specific interventions should be tailored and targeted to people in these high-risk groups to reduce psychological distress in the country. The findings also highlight the need for integrated public health interventions to mediate harmful alcohol use and HIV-positive serostatus on psychological distress.

## Supporting infromation

S1_Data.dta Data used in the analysis. (DTA)
